# 14-3-3 promotes sarcolemmal expression of cardiac Ca_V_1.2 and nucleates isoproterenol-triggered channel superclustering

**DOI:** 10.1073/pnas.2413308122

**Published:** 2025-01-27

**Authors:** Heather C. Spooner, Alexandre D. Costa, Maartje Westhoff, Adriana Hernández-González, Husna Ibrahimkhail, Vladimir Yarov-Yarovoy, Mary C. Horne, Eamonn J. Dickson, Rose E. Dixon

**Affiliations:** ^a^Department of Physiology and Membrane Biology, University of California Davis, Davis, CA 95616; ^b^Department of Anesthesiology and Pain Medicine, University of California Davis, Davis, CA 95616; ^c^Department of Pharmacology, University of California Davis, Davis, CA 95616

**Keywords:** L-type calcium channels, 14-3-3, Rad, ion channel trafficking, *β*-adrenergic receptors

## Abstract

The L-type Ca^2+^ channel (Ca_V_1.2) is essential for cardiac excitation–contraction coupling. To contribute to the inward Ca^2+^ flux that drives Ca^2+^-induced-Ca^2+^-release, Ca_V_1.2 channels must be expressed on the sarcolemma; thus the regulatory mechanisms that tune Ca_V_1.2 expression to meet contractile demand are an emerging area of research. A ubiquitously expressed protein called 14-3-3 has been proposed to affect Ca^2+^ channel trafficking in nonmyocytes; however, whether 14-3-3 has similar effects on Ca_V_1.2 in cardiomyocytes is unknown. 14-3-3 preferentially binds phospho-serine/threonine residues to affect many cellular processes and is known to regulate cardiac ion channels including Na_V_1.5 and the human ether-à-go-go–related gene (hERG) potassium channel. Altered 14-3-3 expression and function have been implicated in cardiac pathologies including hypertrophy. Accordingly, we tested the hypothesis that 14-3-3 interacts with Ca_V_1.2 in a phosphorylation-dependent manner and regulates cardiac Ca_V_1.2 trafficking and recycling. Confocal imaging, proximity ligation assays, superresolution imaging, and coimmunoprecipitation revealed a population of 14-3-3 colocalized and closely associated with Ca_V_1.2. The degree of 14-3-3/Ca_V_1.2 colocalization increased upon stimulation of *β*-adrenergic receptors with isoproterenol. Notably, only the 14-3-3-associated Ca_V_1.2 population displayed increased cluster size with isoproterenol, revealing a role for 14-3-3 as a nucleation factor that directs Ca_V_1.2 superclustering. Isoproterenol-stimulated augmentation of sarcolemmal Ca_V_1.2 expression, Ca^2+^ currents, and Ca^2+^ transients in ventricular myocytes were strengthened by 14-3-3 overexpression and attenuated by 14-3-3 inhibition. These data support a model where 14-3-3 interacts with Ca_V_1.2 in a phosphorylation-dependent manner to promote enhanced trafficking/recycling, clustering, and activity during *β*-adrenergic stimulation.

## Significance

The L-type Ca^2+^ channel, Ca_V_1.2, plays an essential role in excitation–contraction coupling in the heart and in part regulates the overall strength of contraction during basal and fight-or-flight *β*-adrenergic signaling conditions. Proteins that modulate the trafficking and/or activity of Ca_V_1.2 are interesting both from a physiological and pathological perspective, since alterations in Ca_V_1.2 can impact action potential duration and cause arrhythmias. A small protein called 14-3-3 regulates other ion channels in the heart and other Ca^2+^ channels, but how it may interact with cardiac Ca_V_1.2 has never been studied. Examining factors that affect Ca_V_1.2 at rest and during *β*-adrenergic stimulation is crucial for our ability to understand and treat disease and aging conditions where these pathways are altered.

The Ca_V_1.2 subtype of voltage-gated L-type Ca^2+^ channels plays critical roles in excitation–contraction (EC) coupling in smooth and cardiac muscle, excitation–secretion coupling in endocrine cells, and in excitation-transcription coupling in neurons. In cardiomyocytes, depolarization of the sarcolemma by an action potential activates a subset of Ca_V_1.2 channels, allowing Ca^2+^ influx that activates nearby type 2 ryanodine receptors (RyR2) on the junctional sarcoplasmic reticulum (SR). These channels facilitate a graded release of Ca^2+^ from the SR in a process known as Ca^2+^-induced Ca^2+^ release (CICR) and the overall rise in intracellular Ca^2+^ concentration triggers contraction (1). In skeletal muscle, contraction magnitude can be tuned by recruiting more, or less motor units, but in the heart every cardiomyocyte is activated during each beat of the heart thus there are no more cells to be recruited. Instead, the magnitude of cardiac muscle contractility is tuned by the degree of Ca^2+^ influx. Because Ca^2+^ release from the SR is graded, an increase in the trigger Ca^2+^ influx through Ca_V_1.2 also increases the Ca^2+^ transient amplitude and thus contraction magnitude. Accordingly, regulatory pathways, channel auxiliary subunits, and cofactors that can modulate Ca_V_1.2 channel activity and/or sarcolemmal expression are physiologically relevant and thus of particular interest. Furthermore, since alterations in Ca_V_1.2 channel activity can impact action potential duration and promote cardiac arrhythmia (2), alterations in regulatory proteins that modulate channel activity are also interesting from a pathological perspective.

Cardiac Ca_V_1.2 channels are multimeric protein complexes consisting of a pore-forming Ca_V_α_1C_ subunit, an auxiliary Ca_V_β predominantly Ca_V_β_2b_ in cardiomyocytes (3), and a Ca_V_α_2_δ subunit. Depending on the regulatory state of the channel the cardiac Ca_V_1.2 complex may also contain the RGK (Rad, Rem, Rem2, Gem/Kir) protein Rad. These auxiliary proteins associate with the channel complex and have all been found to influence channel expression at the plasma membrane/sarcolemma (4–7). In addition, Rad has been shown to play an essential role in the functional upregulation of Ca_V_1.2 during *β*-adrenergic receptor (*β*-AR) stimulation when PKA-mediated phosphorylation of Rad disrupts its inhibitory interaction with the Ca_V_β auxiliary subunit resulting in increased channel activity (8–12). These findings underscore the importance of identifying and investigating the functional role of Ca_V_1.2 channel cofactors. In this study, we present evidence supporting 14-3-3 as a cardiac Ca_V_1.2 channel interacting protein that affects responsivity to *β*-AR signaling.

14-3-3 is a small (~30 kDa) ubiquitously expressed protein that has many diverse roles in subcellular processes including regulation of transcription, ubiquitination, and chaperoning (13). The numerical name of this protein originated when it was first identified in bovine brain lysates within the 14th elution fraction on a column following DEAE-cellulose chromatography, and for their subsequent electrophoretic mobility on a gel where they migrated to the 3.3 position. There are seven mammalian isoforms of 14-3-3 (β, γ, ε, ζ, η, θ, and σ), each encoded by separate genes *(YWHAB, YWHAG, YWHAE, YWHAZ, YWHAH, YWHAQ*, and *SFN* or *Stratifin*), with 14-3-3ε emerging as the most abundant cardiac isoform at both the protein (14) and mRNA level (15). 14-3-3 isoforms preferentially bind phospho-serine/threonines (16, 17) and are known to modulate the activity, trafficking/cotrafficking, or phosphorylation state of other cardiac ion channels including hERG (18), TASK-1 and TASK-3 (19), Na_V_1.5 (20, 21), Kir2.1 (22), K_ATP_ (15), sarco-endoplasmic reticulum Ca^2+^-ATPase (SERCA) (23), plasma membrane Ca^2+^-ATPase (PMCA) (24, 25), and Na^+^/Ca^2+^ exchanger proteins (NCX) (26). In addition, the trafficking and inactivation kinetics of the neuronal Ca_V_2.2 voltage-gated Ca^2+^ channel is reportedly altered by 14-3-3 (27, 28). Despite the abundance of evidence for the role of 14-3-3 in regulation of other cardiac ion channels and regulators of calcium homeostasis, there is a scarcity of information on the effect of 14-3-3 on Ca_V_1.2 channels, although it is known that membrane-anchored 14-3-3ε (via fusion to a palmitoylated peptide) or membrane-recruitable 14-3-3ε (via fusion to a PKC C1 domain that translocates to the membrane upon PKC activation) affect Ca_V_1.2 activity implying that 14-3-3ε can interact with Ca_V_1.2 (29). Furthermore, 14-3-3 is known to interact with RGK proteins including Rad (30, 31), making it an interesting candidate for phosphorylation-dependent regulation of Ca_V_1.2 trafficking and activity.

Here, we present an examination of the effects of 14-3-3 on multiple layers of Ca_V_1.2 trafficking and regulation. We report that 14-3-3 interacts with transiently transfected Ca_V_1.2 in tsA-201 cells and with endogenous Ca_V_1.2 channels within mouse ventricular myocytes where phosphorylation-dependent 14-3-3 interactions alter Ca_V_1.2 localization and nanoscale redistribution during *β*-AR stimulation. We report that 14-3-3 plays a crucial role in regulating Ca_V_1.2 channel activity and recycling during *β*-adrenergic stimulation. In ventricular myocytes, 14-3-3ε overexpression enhanced both isoproterenol-stimulated *I*_Ca_ amplitude and Ca_V_1.2 plasma membrane expression. Conversely, competitive inhibition of 14-3-3 attenuates isoproterenol-induced increases in *I*_Ca_ and prevents channel recycling and superclustering. Based on our findings, we propose a model wherein 14-3-3 acts as a nucleation factor for Ca_V_1.2 clustering on the sarcolemma.

## Results

### 14-3-3 Associates with Ca_V_1.2 in tsA-201 Cells.

**Fig. 1.**
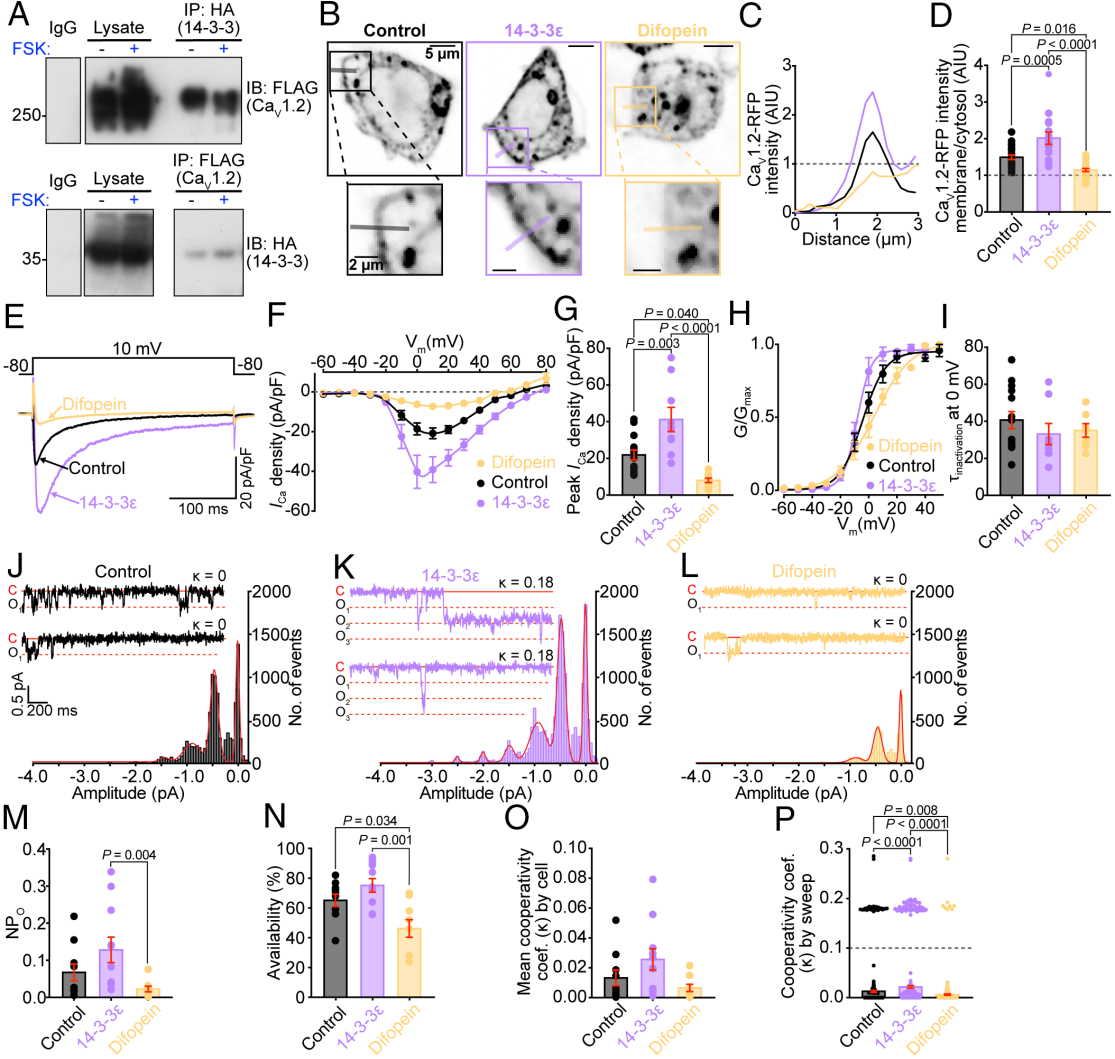
14-3-3 and Ca_V_1.2 co-immunoprecipitate and 14-3-3 levels regulate Ca_V_1.2 surface expression and whole cell calcium currents in tsA-201 cells. (*A*) IBs showing IgG control, lysate, and 14-3-3 IP lanes probed for Ca_V_1.2 and Ca_V_1.2 IP lanes probed for 14-3-3 in tsA-201 lysates treated with FSK (+), or with vehicle alone (−). (*B*) Representative confocal images showing Ca_V_1.2-RFP localization in control, 14-3-3ε overexpressing, or difopein-expressing tsA-201 cells. (*C*) Plot profiles of membrane/cytosolic RFP intensity across the line regions-of-interest indicated in *B*. (*D*) histogram summarizing the ratio between membrane and cytosolic Ca_V_1.2-RFP intensity (control: *n* = 22; 14-3-3ε: *n* = 15; difopein: *n* = 20). (*E*) Representative whole-cell currents elicited from transfected tsA-201 cells from each condition. (*F*) I-V plots and (*G*) histogram summarizing peak *I_Ca_* density (control: *n* = 14; 14-3-3ε: *n* = 9; difopein: *n* = 9). (*H*) Voltage dependence of the normalized conductance (G/Gmax) fit with Boltzmann functions (control: *n* = 13; 14-3-3ε: *n* = 8; difopein: *n* = 7). (*I*) Histogram showing time constant (τ) of inactivation at 0 mV. (*J*–*L*) All-points histograms with *Inset* representative *i_Ca_* traces with corresponding cooperativity coefficient (κ) for control (*J*, *n* = 10, 474 sweeps), 14-3-3ε (*K*, *n* = 11, 481 sweeps), and difopein cells (*L*, *n* = 9, 448 sweeps). Amplitude histograms were fit with multicomponent Gaussian functions (red lines). (*M*–*P*) Summary data of NP_O_ (*M*), channel availability (*N*), average cooperativity coefficient (κ) per cell (*O*), and κ per sweep (*P*) (control: *n* = 10; 14-3-3ε: *n* = 11; difopein: *n* = 9). Data were analyzed using one-way ANOVAs (*D*, *G*, *I*, and *N*), or Kruskal–Wallis tests (*M*, *O*, and *P*) with multiple comparison post hoc tests. Error bars indicate SEM.

To determine whether 14-3-3 associates with Ca_V_1.2, we performed coimmunoprecipitation (co-IP) assays to probe for Ca_V_1.2-14-3-3 interactions. We chose to focus on 14-3-3ε based on RT-PCR experiments showing 14-3-3ε has the highest mRNA expression in mouse and rat ventricular myocytes (15), and a study that found a membrane-targeted version of 14-3-3ε was able to modify Ca_V_1.2 activity in HEK-293 cells or cultured murine dorsal root ganglion neurons, although the function and nature of this potential interaction and whether it applied to native 14-3-3 protein was not tested (29). We initially adopted a reductionist system and performed IPs on whole cell lysates from tsA-201 cells transiently transfected with FLAG-tagged Ca_V_α_1C_, auxiliary Ca_V_β_2b_-cerluean and Ca_V_α_2_δ, and HA-tagged 14-3-3ε. The association of 14-3-3 with Ca_V_1.2 was assessed by immunoprecipitation (IP) of the tagged-proteins from the lysate followed by sodium dodecyl sulfate–polyacrylamide gel electrophoresis (SDS-PAGE) fractionation of the complexes and immunoblot (IB) analysis of the fractionated proteins. To assess the association of Ca_V_1.2 with 14-3-3ε, anti-HA (14-3-3ε) immunocomplexes were probed with an anti-FLAG (Ca_V_α_1C_) antibody (Fig. 1 *A*, *Top*). To determine whether the reverse, i.e. pull-down of Ca_V_1.2 would also bring down 14-3-3ε associated with it, the reciprocal IP using anti-FLAG followed by IB with anti-HA was performed (Fig. 1 *A*, *Bottom*). In both cases, appreciable amounts of the putative complex partner were observed, confirming association of Ca_V_1.2 with 14-3-3ε in these cells.

14-3-3 preferentially binds to phosphorylated serine or threonine residues on various proteins via electrostatic interactions (16, 32–34). In hERG K^+^ channels, another cardiac ion channel, phosphorylation-dependent interactions with 14-3-3 have been reported to shield phosphorylated residues from phosphatases thus prolonging *β*-AR stimulation of hERG activity (18). Recently, a similar mechanism also has been shown for phospholamban, the small regulatory protein that confers PKA regulation on the cardiac SR Ca^2+^ pump SERCA (23). To determine whether Ca_V_1.2-14-3-3 interactions can occur in a phosphorylation-dependent manner, co-IPs were performed on tsA-201 cell lysates, transfected as described above and treated with the adenylyl cyclase activator forskolin (FSK; 10 µM for 8 min). No obvious FSK-stimulated increase in Ca_V_1.2-14-3-3 interactions was detected (Fig. 1*A*), however we cannot rule out that HA-14-3-3 is already associated with phosphorylated residues on Ca_V_1.2 in the control (no FSK) lysates and that our assay is not sensitive enough to detect additional association with FSK, or that direct 14-3-3-Ca_V_1.2 interactions are dependent on phosphorylation by a kinase not stimulated by FSK. It is also important to note that while tsA-201 cells do not endogenously express Ca_V_1.2, they do express significant amounts of 14-3-3 (35). Thus, the interactions observed here are likely an underestimation since they detect only overexpressed HA-tagged 14-3-3 in complex with Ca_V_1.2, and are blind to endogenous 14-3-3-Ca_V_1.2 complexes.

### Surface Expression of Ca_V_1.2 Is Promoted by 14-3-3.

Having established that 14-3-3 and Ca_V_1.2 associate with one another, we next investigated whether these interactions could promote surface trafficking of Ca_V_1.2 channels as has been reported for N-type Ca_V_2.2 channels (28). To test that idea, we used tsA-201 cells transiently transfected with the channel [Ca_V_α_1C_-red fluorescent protein (RFP)] and auxiliary subunits (Ca_V_β_2b_ and Ca_V_α_2_δ). In some experiments cells were additionally transfected with 14-3-3ε to achieve overexpression, or with a plasmid encoding an EYFP-tagged 14-3-3 inhibitor called difopein (for dimeric fourteen-three-three peptide inhibitor; a doublet of R18 inhibitory peptide; pSCM138) to compete for 14-3-3 binding or as a control, the plasmid pSCM174 encoding an inactivated form of the difopein peptide (an EYFP-fused doublet of R18 containing D12K and E14K mutations) (36). After 48 h, we measured surface localization of RFP-tagged Ca_V_1.2 using confocal microscopy (Fig. 1 *B*–*D*). The localization of Ca_V_1.2 at or close to the plasma membrane was significantly reduced in cells cotransfected with difopein when compared to no difopein or inactivated difopein control cells (Fig. 1 *B*–*D* and *SI Appendix,* Fig. S1 *A* and *B*). Channels appeared to be stuck in intracellular compartments in difopein-expressing cells implying impaired trafficking processes. We found no significant difference in Ca_V_1.2 surface localization in the no difopein controls as compared to those expressing inactive difopein (*SI Appendix,* Fig. S1 *A* and *B*) and thus we pooled those cells in all subsequent analyses. To determine whether this role in Ca_V_1.2 trafficking facilitation extended beyond 14-3-3ε and applied to other 14-3-3 isoforms, we repeated this experiment with multiple 14-3-3 isoforms (*SI Appendix,* Fig. S1 *B* and *C*). While trending increases in Ca_V_1.2 plasma membrane localization were observed with every 14-3-3 isoform examined, the only statistically significant change was with 14-3-3ε, providing additional confidence in our decision to focus on 14-3-3ε.

If 14-3-3 does promote surface expression of Ca_V_1.2 in tsA-201 cells then two logical predictions are i) 14-3-3 overexpression should increase whole cell calcium current (*I_Ca_*) and ii) disruption of 14-3-3–Ca_V_1.2 interactions with difopein should decrease the number of functional channels at the surface thus reducing *I_Ca_*. To test those predictions, we measured *I_Ca_* in tsA-201 cells transfected as described above and saw a 1.88 ± 0.30-fold increase in *I_Ca_*with 14-3-3ε overexpression, and very little current in cells with difopein (Fig. 1 *E*–*G*). We did not observe significant changes in the voltage at half maximal activation (V_1/2_), a measure of voltage dependence of activation or in inactivation kinetics with any perturbation (Fig. 1 *H* and *I* and *SI Appendix,* Table S1). Since *I*_Ca_ is given by the product of the number of active channels, their open probability, and their unitary current (*i*_Ca_) amplitude (*I_ca_* = *N* × *P_o_* × *i_ca_*) we sought to elucidate which of these parameters were altered to explain the changes in *I*_Ca_ with 14-3-3ε overexpression or competitive inhibition. Cell-attached patch single-channel recordings were thus performed with currents evoked using step depolarizations to −30 mV with Ca^2+^ as the charge carrier. These experiments revealed that control (or inactivated difopein expressing), 14-3-3ε overexpressing, and difopein expressing cells all had similar unitary current (*i*_Ca_) amplitudes (control −0.482 ± 0.018 pA, 14-3-3ε −0.500 ± 0.014 pA, difopein −0.444 ± 0.013 pA; Fig. 1 *J*–*L*). A trending increase in single-channel activity (*NP*_o_) occurred with 14-3-3ε overexpression, while difopein significantly reduced *NP*_o_ (Fig. 1*M*). Thus, in the absence of changes in the unitary conductance, the effects of 14-3-3 on *I*_Ca_ appear to occur in part due to alterations in single-channel activity (*P*_o_), and to changes in the number of active channels (*N*).

### 14-3-3 Augments Cooperative Interactions of Ca_V_1.2 Channels.

All-points histograms compiled from multiple cells revealed that multichannel openings were more likely in 14-3-3ε overexpressing cells (Fig. 1 *J*–*L*). In contrast, we observed reduced channel availability (likelihood of observing at least one event/sweep) in difopein expressing cells (Fig. 1*N*). With our results supporting a role for 14-3-3 in promoting surface expression and availability of Ca_V_1.2 channels, we reasoned this could have implications for their cooperative gating. This gating modality occurs when Ca_V_1.2 channels physically interact within clusters, where they influence each other’s activity so that the opening of one channel increases the likelihood of opening of the attached neighboring channels resulting in an amplification of Ca^2+^ influx (37–39). Overexpression of 14-3-3ε significantly increased the slope steepness of the Boltzmann function used to fit the G/G_max_ data compared to control while a shallower slope was observed in the difopein group (*SI Appendix,* Table S1) suggesting 14-3-3ε increased cooperativity of the channels while difopein decreased it. To further quantify cooperativity within our *i*_Ca_ data, we applied a coupled Markov chain model (37, 39–42). This model assigns a cooperativity coefficient (κ) to each sweep with a value of 0 indicating exclusively independently gating channels and a value of 1 indicating exclusively cooperatively gating channels underlying the currents. Upon examination of cellular averages, 14-3-3ε overexpressing cells had a trending increase in κ, while difopein expressing cells displayed a slight decrease in κ (Fig. 1*O*) compared to controls. Using κ ≥ 0.1 as a threshold for cooperative gating, closer inspection of the data revealed that cooperative openings occurred more frequently in 14-3-3ε overexpressing cells and less frequently in difopein expressing cells compared to controls (Fig. 1*P*). Taken together these data suggest a possible role for 14-3-3ε in promoting cooperative interactions between Ca_V_1.2 channels when expressed in tsA-201 cells.

### 14-3-3 Cannot Drive Surface Trafficking of Ca_V_α_1C_ without Ca_V_β.

Given the clear Ca_V_1.2 trafficking enhancing effects of 14-3-3ε, we wondered whether 14-3-3ε overexpression could drive trafficking of the Ca_V_α_1C_ subunit to the plasma membrane without a requirement for coexpression of Ca_V_β auxiliary subunits. Indeed a prior study found 14-3-3τ overexpression sufficient to force trafficking of Ca_V_α_1B_ (pore-forming subunit of Ca_V_2.2) in tsA-201 cells without coexpression of any auxiliary subunits (28); to test whether a similar mechanism exists for Ca_V_1.2, we overexpressed 14-3-3ε with Ca_V_α_1C_ and Ca_V_α_2_δ without the Ca_V_β subunit and found that 14-3-3ε overexpression was not sufficient to drive trafficking of Ca_V_1.2 independent of the Ca_V_β subunit (*SI Appendix,* Fig. S2). Rather, 14-3-3ε appears to play a coregulatory role in Ca_V_α_1C_ trafficking since its overexpression enhanced channel surface expression only in the presence of Ca_V_β.

### *β*-AR Stimulation Enhances 14-3-3–Ca_V_1.2 Colocalization on the T-Tubule Sarcolemma.

**Fig. 2.**
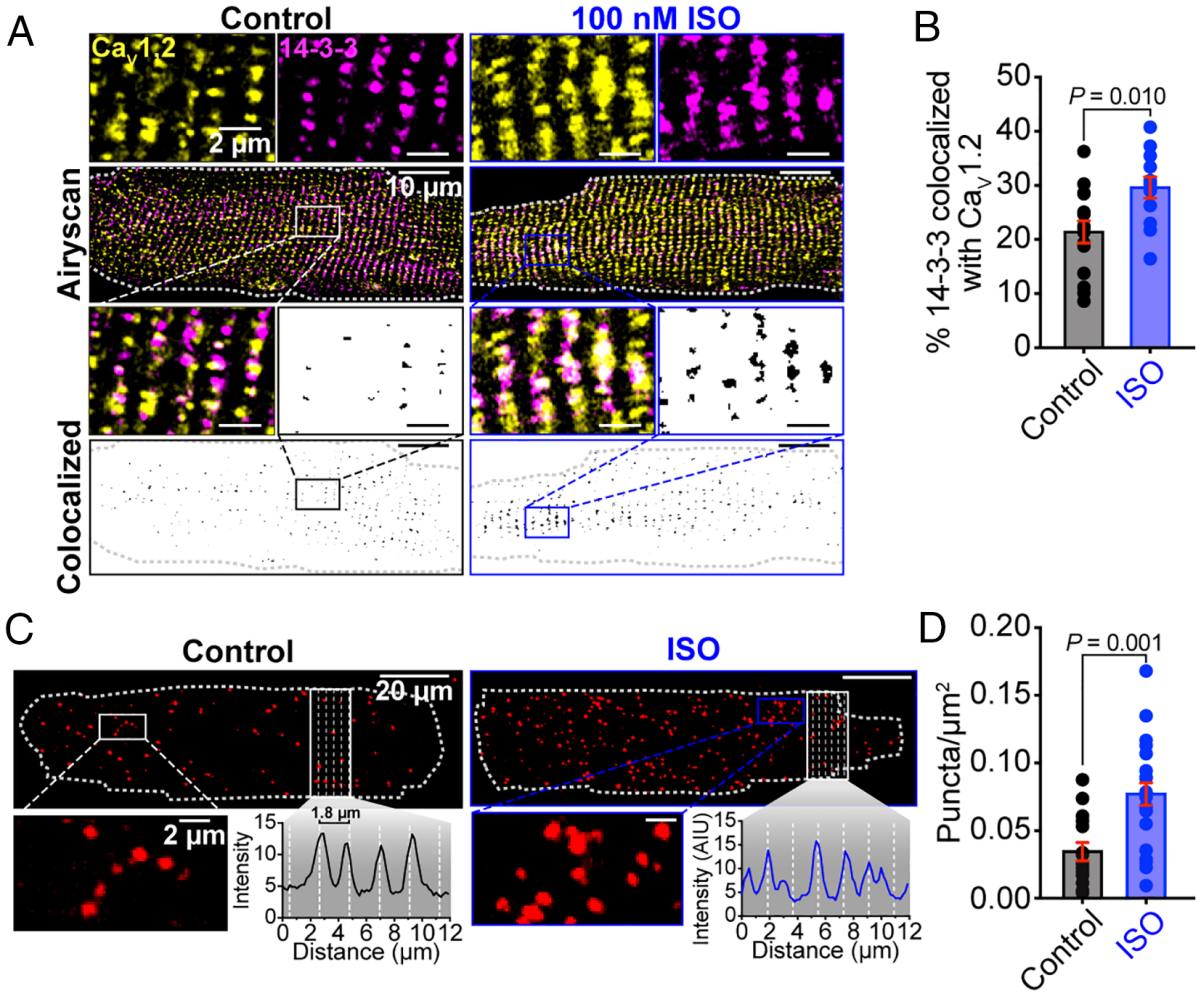
14-3-3 and Ca_V_1.2 colocalize in cardiomyocytes, and isoproterenol stimulates an increase in this colocalization. (*A*) Representative Airyscan images of myocytes with and without ISO immunostained against Ca_V_1.2 and 14-3-3. (*Bottom*) Binary colocalization maps showing regions of complete pixel overlap between signals. (*B*) Histogram summarizing % colocalization between 14-3-3 and Ca_V_1.2 in myocytes with and without ISO (control: *N* = 4, *n* = 15; ISO: *N* = 3, *n* = 12). (*C*) Representative images of PLA between 14-3-3 and Ca_V_1.2 in myocytes with and without ISO with inset intensity plot profiles showing approximate z-line localization of proximity sites (puncta). (*D*) Puncta per μm^2^ in myocytes with and without ISO (control: *N* = 3, *n* = 15; ISO: *N* = 3, *n* = 22). Data were analyzed using unpaired Student’s *t* tests. Error bars indicate SEM.

Given our finding that endogenous 14-3-3 co-IPs with Ca_V_1.2 in tsA-201 cell lysates, we next investigated the relative localization of the two proteins in isolated ventricular myocytes. Although 14-3-3 is a cytosolic protein, it is known to associate with many membrane proteins and has been shown to accumulate in areas where it has strong regulatory effects such as synapses (43) and intercalated disks (20). We examined 14-3-3 and Ca_V_1.2 distribution and colocalization in freshly isolated adult mouse ventricular myocytes using Airyscan superresolution imaging of cells immunolabeled for Ca_V_1.2 and 14-3-3 in control and isoproterenol (ISO)-stimulated conditions (Fig. 2*A*). In methanol and Triton X-100 permeabilized cells, much of the cytosolic 14-3-3 is lost but much of the membrane protein-bound 14-3-3 appeared to be retained, and a striking z-line 14-3-3 localization pattern was evident (Fig. 2*A*). A similar distribution was observed in paraformaldehyde-fixed cells permeabilized with either saponin or Triton X-100 (*SI Appendix,* Fig. S3). In unstimulated (no ISO) control myocytes, 20.92 ± 1.99% of the endogenous 14-3-3 was found colocalized with Ca_V_1.2 at this resolution (Fig. 2 *A* and *B*). Acute ISO (100 nM for 8 min) stimulation of isolated ventricular myocytes prior to fixation resulted in a significant increase in 14-3-3–Ca_V_1.2 colocalization (from 21.42 ± 2.06% in control, to 29.59 ± 1.99% in ISO; Fig. 2 *A* and *B*). This ISO-stimulated increase in 14-3-3–Ca_V_1.2 interactions was further confirmed using proximity ligation assays (PLA; Fig. 2 *C* and *D*). In this technique, ventricular myocytes were immunostained with anti-Ca_V_1.2 and anti-14-3-3 primary antibodies coupled to species-specific DNA primer tagged secondary antibodies. When the two sets of primer-tagged probes (Ca_V_1.2-PLUS and 14-3-3-MINUS) come within 40 nm of one another, they hybridize. Following ligation to form circular DNA, amplification of the DNA and addition of a fluorescent detection reagent allows visualization of fluorescent puncta as a readout at the sites of proximity (44). Accordingly, we observed 23.35% more puncta on average in ISO-stimulated myocytes compared to unstimulated controls (Fig. 2*D*). Together with the Airyscan data, these results suggest that 14-3-3 associates with Ca_V_1.2 channels on the t-tubule sarcolemma and that the association is enhanced upon activation of the *β*-AR signaling cascade.

### 14-3-3 Interactions Affect the Nanoscale Distribution and Clustering of Ca_V_1.2 Channels.

**Fig. 3.**
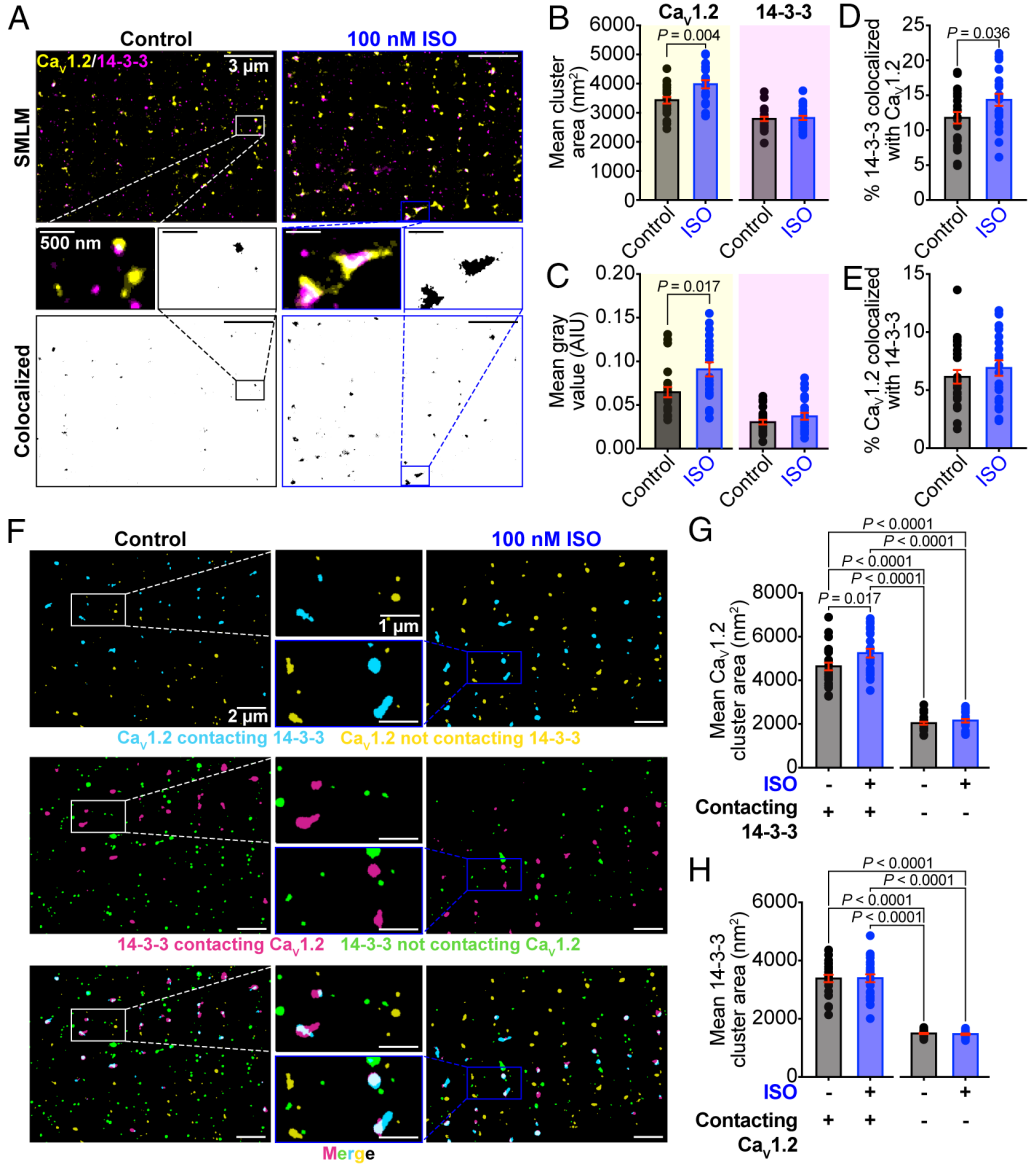
14-3-3 acts as a nucleation factor for *β*-AR stimulated Ca_V_1.2 superclustering. (*A*) SMLM maps of cardiomyocytes immunostained against Ca_V_1.2 and 14-3-3 in myocytes with or without ISO-stimulation. (*B* and *C*) Histograms summarizing mean Ca_V_1.2 channel cluster area (control: *N* = 3, *n* = 21; ISO: *N* = 3, *n* = 20), 14-3-3 channel cluster area (control: *N* = 4, *n* = 25; ISO: *N* = 4, *n* = 25), Ca_V_1.2 gray value (control: *N* = 4, *n* = 23; ISO: *N* = 4, *n* = 22), and 14-3-3 gray value (control: *N* = 4, *n* = 25; ISO: *N* = 4, *n* = 25). (*D*) Histograms summarizing % colocalization between 14-3-3 and Ca_V_1.2, and (*E*) % colocalization between Ca_V_1.2 and 14-3-3 in myocytes with and without ISO (control: *N* = 4, *n* = 24; ISO: *N* = 4, *n* = 22). (*F*) SMLM maps showing 14-3-3 clusters in contact (magenta) and not in contact (green) with Ca_V_1.2, and Ca_V_1.2 clusters in contact (teal) and not in contact (yellow) with 14-3-3. (*G*) Histogram summarizing mean Ca_V_1.2 channel cluster area (control: *N* = 4, *n* = 25; ISO: *N* = 4, *n* = 24) and (*H*) 14-3-3 channel cluster area (control: *N* = 3, *n* = 24; ISO: *N* = 4, *n* = 24). Data were analyzed using unpaired Student’s *t* tests (*B*, *D*, and *E*), and Mann–Whitney tests (*C*). Data in *G* and *H* were analyzed using two-way ANOVAs with multiple comparison post hoc tests. Error bars indicate SEM.

We have previously reported that Ca_V_1.2 recycling from endosomal reservoirs is enhanced by acute *β-*AR stimulation with ISO (45–48). This enhanced recycling leads to mobilization of endosomal Ca_V_1.2 channels to the t-tubule sarcolemma where they form larger clusters. Thus, we hypothesized that the increase in colocalization between 14-3-3 and Ca_V_1.2 with ISO could simply occur because of that increased t-tubular localization and clustering of Ca_V_1.2. We tested this hypothesis by performing single molecule localization microscopy (SMLM; lateral resolution of ~30 nm) to examine the nanoscale distribution, clustering, and colocalization of 14-3-3 and Ca_V_1.2 in control and ISO-stimulated myocytes. At this enhanced resolution the z-line pattern of 14-3-3 and Ca_V_1.2 localization remained evident and discrete areas of colocalization of the two proteins were observed as in the Airyscan imaging experiments. Consistent with previous findings, Ca_V_1.2 clustering and expression along the t-tubule sarcolemma significantly increased with ISO (Fig. 3 *A*–*C*). Although we expected 14-3-3 cluster size and expression to concurrently increase, neither parameter was significantly increased after ISO (Fig. 3 *A*–*C*). Rather than cotrafficking with the channel, this suggests that 14-3-3 may be acting as an organizing point or nucleation factor for Ca_V_1.2 superclustering. In these experiments, we also saw a significant increase in the percent of 14-3-3 colocalized with Ca_V_1.2, but not in the percent of Ca_V_1.2 colocalized with 14-3-3 (Fig. 3 *D* and *E*; note the lower colocalization in SMLM experiments relative to Airyscan are likely due to the difference in resolution as there is less spatial broadening/blur of the signal in SMLM). Accordingly, we measured the Ca_V_1.2 cluster size versus their distance to 14-3-3 and found that not only were the clusters in contact with 14-3-3 larger in control conditions, but only this population increased after ISO (Fig. 3 *F*–*H*). Collectively these results suggest that ISO-stimulated recycling of Ca_V_1.2 channels selectively occurs at sites on the t-tubule sarcolemma where 14-3-3 is already present.

### Phosphorylation-Dependent 14-3-3 Binding Sites on Ca_V_1.2.

With accumulating evidence pointing toward phosphorylation augmented 14-3-3 binding to Ca_V_1.2, we next mapped potential binding sites for 14-3-3 on Ca_V_1.2 by overlaying known phosphorylation sites (49) with the 14-3-3 binding motifs using 14-3-3-Pred, a web-based tool that predicts 14-3-3 binding sites in proteins (33) (*SI Appendix,* Fig. S4*A*). The rabbit gene was used for numbering for Ca_V_α_1C_ and Ca_V_β_2b_, but equivalent sites also exist for the human and mouse genes. 14-3-3 dimers have two amphipathic binding grooves that can permit binding of pairs of phosphorylated residues located >15 amino acids apart (32). Two optimal consensus binding motifs for 14-3-3 have been identified, mode I R(S/X)X(pS/T)XP, and Mode II RX(F/Y)X(pS/T)XP where X is any amino acid and pS/T denotes phosphorylated serine or threonine (32). An additional third mode allows a lower affinity binding of 14-3-3 to the final few amino acids at a protein C-terminal end accordingly the mode III motif is p(S/T)X_1−2_-COOH (50). However, several noncanonical 14-3-3 binding motifs have been reported that can subtly or even quite wildly deviate from these, including some that do not require phosphorylation (51). There are many phosphorylation sites on both the Ca_V_α_1C_ pore-forming subunit of Ca_V_1.2 and the auxiliary Ca_V_β_2b_ subunit (49), but only five along the C-terminal tail of the Ca_V_α_1C_ subunit, and three in the Ca_V_β_2b_ subunit were identified as being putative 14-3-3 binding sites. Of interest, an additional 14-3-3 binding site was identified at the extreme distal C-terminus of the Ca_V_α_1C_ subunit which allows for the possibility of mode III binding, although this serine is not a known phosphorylation site. In this binding mode only one of the 14-3-3 dimer protein binding pockets is occupied, making the interaction with the client protein unstable. These interactions can be dramatically stabilized by the fungal toxin fusicoccin A (FC-A) which occupies the vacant binding pocket in the 14-3-3 dimer to stabilize the interaction (52). We reasoned that addition of FC-A to our assay would allow us to resolve unstable mode III interactions if they were present between 14-3-3 and Ca_V_1.2. If the putative 14-3-3 biding site on the extreme distal C-terminal is significantly involved in the phosphorylation-dependent binding of 14-3-3 to Ca_V_1.2 then we predicted that stabilization of that interaction with FC-A would result in a more prominent FSK-stimulated interaction in our co-IPs. To test that, we performed co-IPs in the presence of FC-A; however, we observed no appreciable difference between FC-A treated and untreated samples, with no additional FSK effects (*SI Appendix,* Fig. S4*B*). These data suggest that this Ca_V_α_1C_ distal C-terminal site is not a major binding site for 14-3-3 to Ca_V_1.2 but it remains to be determined which of the other identified sites are essential for these interactions.

### Modeling of Ca_V_1.2 Interaction with 14-3-3 Using AlphaFold 3 (AF3).

Seeking insight into possible interaction sites between the Ca_V_1.2 complex and 14-3-3ε, we utilized AF3 (53) to model potential interactions between a dimer of 14-3-3ε and i) phospho-Ca_V_α_1C_; ii) phospho-Ca_V_β_2_; and iii) phospho-Rad as described in the *Materials and Methods*. AF3 is a diffusion-based deep neural network capable of predicting the biological structure of protein complexes with nucleic acids, small molecules, ions, and posttranslational modifications, including protein phosphorylation. The AF3-predicted model of rabbit and human Ca_V_1.2 closely resembles cryoelectron microscopy structure of human Ca_V_1.2 (PDB: 8we6) (54), with a root mean square deviation (RMSD) between Cα atoms of ~2.8 Å and ~3.2 Å, respectively, over the experimentally resolved regions of the human Ca_V_1.2 structure (*SI Appendix,* Fig. S5*A*). Similarly, the AF3 model of a human 14-3-3ε dimer closely agreed with the published X-ray structure (PDB: 7c8e) (55), with RMSD ~0.9 Å (*SI Appendix,* Fig. S5*B*). AF3 per-residue predicted local distance difference test (pLDDT) confidence scores in the rabbit Ca_V_1.2 model of the C-terminal region varied from very low (21) to low (50-70) to good (71) levels (*SI Appendix,* Fig. S5 *E* and *F*), corresponding to the comparatively less ordered nature of the C-terminal region which has so far not been fully resolved. The model failed to predict any association between Ca_V_1.2 α_1C_ and 14-3-3ε in the absence of phosphorylation. In contrast, the addition of phosphate groups at S1700 and S1928 allowed interactions of Ca_V_α_1C_ with two 14-3-3ε molecules, in the conformation expected of a dimer (*SI Appendix,* Fig. S5 *C* and *D*). Notably, pLDDT confidence in the C-terminal regions containing phosphorylation sites was very low (41-46) at pS1700 and pS1928 in the AF3 model of rabbit Ca_v_1.2, and low (~52) at pS1718 and pS1981 in the AF3 model of human Ca_v_1.2 (*SI Appendix,* Fig. S5 *E* and *F*). Despite these limitations, this AF3-generated model predicts a phosphorylation-dependent association between a dimer of 14-3-3ε and the C-terminal tail of Ca_V_1.2 α_1C_ via pS1928 and pS1700, two residues known to be phosphorylated during *β*-AR stimulation in vivo.

We next examined the potential for 14-3-3ε–phospho-Ca_V_β interactions. All possible pairwise combinations of Ca_V_β phosphorylation were modeled, and the best models were selected based on the pLDDT scores. AF3 identified potential interactions between 14-3-3ε and several phosphorylated residue pairs on Ca_V_β_2_ (pS514 and pT554; pS514 and pS630; and pT554 and pS630; *SI Appendix,* Fig. S6). These sites are homologous to S486, T526, and S602 on rabbit Ca_V_β_2b_ depicted in *SI Appendix,* Fig. S4 and identified as putative 14-3-3 binding sites. Interestingly, AF3 also predicts that 14-3-3ε can interact with phosphorylated Rad (*SI Appendix,* Fig. S7). Rad is an RGK protein that forms part of the Ca_V_1.2 complex, interacting with Ca_V_β to dampen Ca_V_1.2 channel activity but becoming dislodged from Ca_V_β upon its phosphorylation by PKA during *β*-adrenergic signaling, thus relieving the suppression of channel activity (8–12). Prior biochemical studies revealed that 14-3-3 binds to both Rad and Rem in a phosphorylation-dependent manner and identified some phospho-serine residues on the Rad/Rem C-terminal as forming part of the binding motif (31, 56). AF3 predicts that both of these same phospho-serine residues pS39 and pS301 on Rad are important for the 14-3-3ε–phospho-Rad interaction. These AF3 models and analyses reveal multiple possible sites of interaction of 14-3-3ε with the Ca_V_1.2 channel complex and future studies should determine their functional importance.

### Acute 14-3-3 Inhibition Impairs ISO-Stimulated Ca_V_1.2 Channel Recycling.

**Fig. 4.**
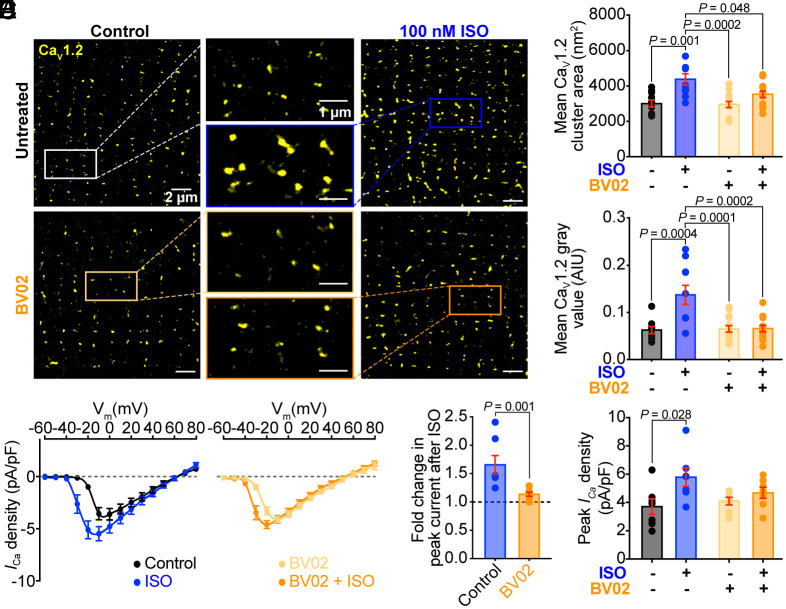
Acute 14-3-3 inhibition abrogates *β*-AR stimulated Ca_V_1.2 superclustering in isolated cardiomyocytes. (*A*) Representative SMLM localization maps of cardiomyocytes immunostained against Ca_V_1.2 in untreated (*Top Left*), 100 nM ISO treated (*Top Right*), 50 μM cell permeable 14-3-3 inhibitor BV02 treated (*Bottom Left*), and combined ISO-BV02 treated (*Bottom Right*) conditions. (*B*) Histograms summarizing mean Ca_V_1.2 channel cluster area (*N* = 3 for all groups, control: *n* = 10; ISO: *n* = 10; BV02: *n* = 14; ISO-BV02: *n* = 13), and (*C*) Ca_V_1.2 gray value (*N* = 3 for all groups, control: *n* = 9; ISO: *n* = 10; BV02: *n* = 14; ISO-BV02: *n* = 13). (*D*) I-V plots, (*E*) fold change in peak *I_Ca_* density after ISO, and (*F*) histogram summarizing peak *I_Ca_* density (untreated: *N* = 4; BV02: *N* = 3, *n* = 7 for all groups). Data were analyzed using two-way ANOVAs with multiple comparison post hoc tests (*B*, *C*, and *F*), or Mann–Whitney tests (*E*). Error bars indicate SEM.

We hypothesized that a phosphorylation-dependent association between 14-3-3 and Ca_V_1.2 in cardiomyocytes that impacts ISO-stimulated channel superclustering would have an impact on the functional responsivity and redistribution of Ca_V_1.2 upon *β*-AR activation. To begin addressing that, we first tested the role of endogenous 14-3-3 in basal Ca_V_1.2 channel clustering and ISO-stimulated superclustering, using ventricular myocytes acutely treated with the cell-permeable 14-3-3 inhibitor BV02 (50 µM, for 8 min, at 37 °C). Cells were subsequently immunostained and imaged using SMLM. Results revealed that acute BV02 treatment did not significantly affect basal cluster size, localization, or expression of Ca_V_1.2 compared to PBS-treated controls however it significantly attenuated the ISO-stimulated superclustering response (Fig. 4 *A*–*C*). Consistent with the SMLM results, whole cell patch clamp recordings of *I*_Ca_ in freshly isolated ventricular myocytes treated with BV02 at room temperature revealed no effect of the 14-3-3 inhibitor on basal current amplitude but a significant attenuation of the ISO-induced increase in *I*_Ca_ compared to controls (Fig. 4 *D*–*F*). These data support a model where 14-3-3 plays a critical role in ISO-stimulated Ca_V_1.2 endosomal recycling and/or insertion of channels into the membrane but does not overtly affect the basal sarcolemmal Ca_V_1.2 population at least on this acute timescale.

### 14-3-3 Overexpression or Prolonged Inhibition Alters Ca_V_1.2 Responsivity to *β*-Adrenergic Stimulation in Cardiomyocytes.

We next examined the effects of more prolonged alterations in 14-3-3 in isolated cardiomyocytes. Cells were cultured for up to 48 h to allow adenoviral transduction with either RFP (control), 14-3-3ε-mRuby, or difopein-YFP. Given that primary cultures of adult mouse cardiomyocytes often result in t-tubule remodeling, we initially performed wheat germ agglutinin staining to examine the effects of i) the 2-d culture period, ii) 14-3-3ε overexpression, and iii) difopein on t-tubule organization. Our blebbistatin-supplemented culture media preserved the integrity of the t-tubule network over this time period (*SI Appendix,* Fig. S8). Furthermore, neither 14-3-3ε overexpression nor difopein caused any significant structural alterations. Additionally, measurements of cell capacitance across all three cohorts revealed no significant changes in cell size, further corroborating that the culture period did not significantly alter the cytoarchitecture.

**Fig. 5.**
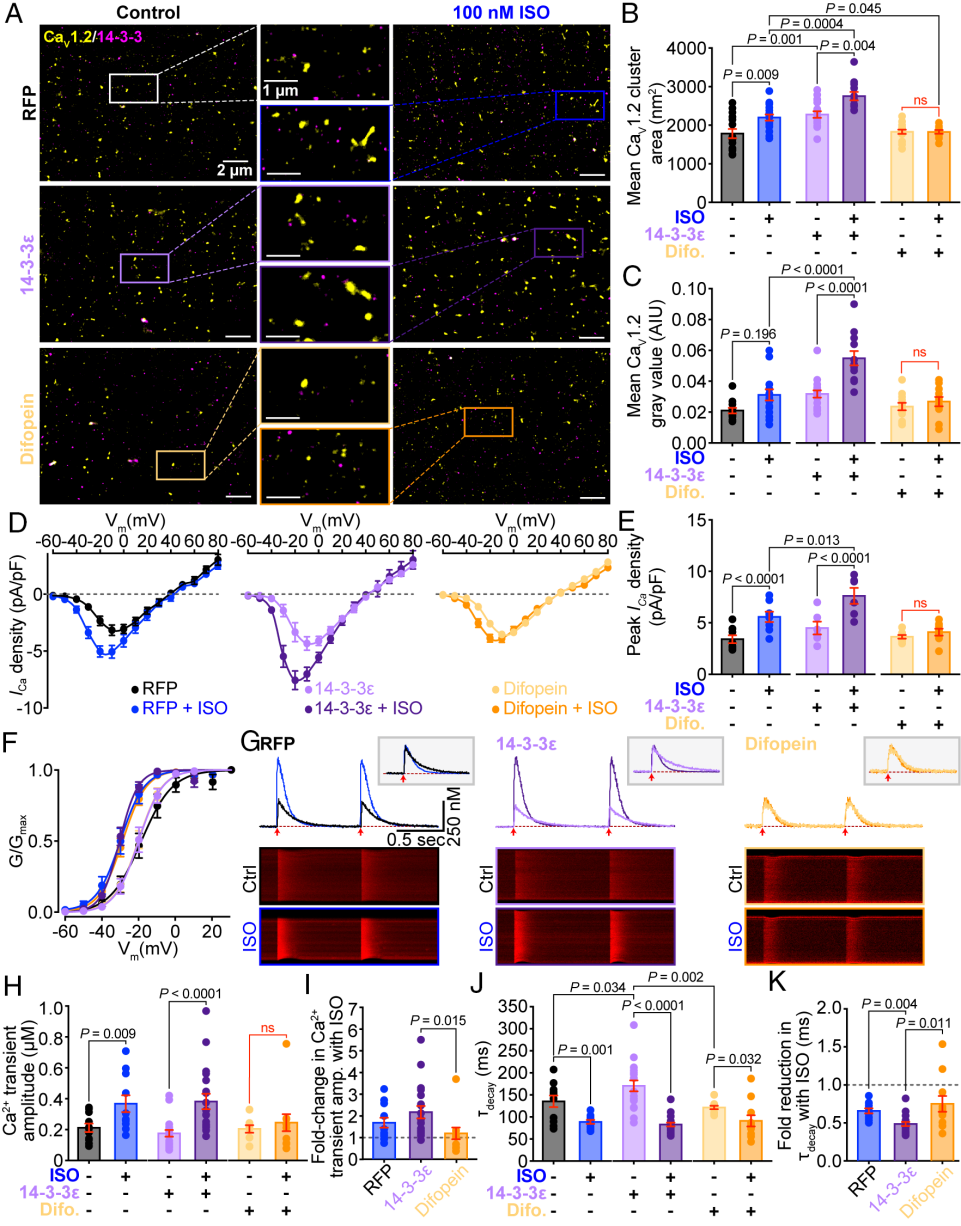
14-3-3 overexpression enhances basal Ca_V_1.2 density, cluster size, and *I_Ca_*, with a preserved *β*-AR response, while 14-3-3 inhibition prevents the *β*-AR stimulated superclustering and increased *I_Ca_* response. (*A*) Representative SMLM localization maps of isolated cardiomyocytes cultured for 48 h with Ad-RFP (*control*), Ad-14-3-3ε-mRuby, or Ad-EYFP-difopein and immunostained against Ca_V_1.2 and 14-3-3 with and without ISO treatment. (*B* and *C*) Histograms summarizing mean Ca_V_1.2 channel cluster area (*N* = 4 for all groups, RFP control: *n* = 15, ISO: *n* = 18; 14-3-3ε control: *n* = 17, ISO: *n* = 12; difopein control: *n* = 16, ISO: *n* = 12), and Ca_V_1.2 gray area (*N* = 4 for all groups, RFP control: *n* = 12, ISO: *n* = 17; 14-3-3ε control: *n* = 17, ISO: *n* = 12; difopein control: *n* = 16, ISO: *n* = 11). (*D*) Plots showing the voltage dependence of *I*_Ca_ density (RFP: *N* = 6, *n* = 8; 14-3-3ε: *N* = 5, *n* = 6; difopein: *N* = 5, *n* = 8). (*E*) Histograms showing peak *I_Ca_* density, and (*F*) plots showing the voltage dependence of activation for cells shown in *D*. (*G*) Example Ca^2+^ transient recorded before (black) and after ISO (blue) from paced cardiomyocytes with *Inset* normalized traces to show decay. (*H*) Ca^2+^ transient amplitude, (*I*) Fold change in Ca^2+^ transient amplitude after ISO, (*J*) Time constant of decay τ, and (*K*) Fold change in τ_decay_ (*N* = 3 for all groups, RFP: *n* = 11; 14-3-3ε: *n* = 19; difopein: *n* = 11). Data were analyzed using two-way ANOVAs (*B*, *C*, *E*, *H*, and *J*), one-way ANOVAs (*K*), or Kruskal–Wallis tests (*I*) with multiple comparison post hoc tests. Error bars indicate SEM.

Subsequent SMLM experiments were performed on unstimulated and ISO-stimulated cohorts of cells immunolabeled against Ca_V_1.2 and 14-3-3. SMLM performed on cultured cells transduced with RFP confirmed that a robust and significant ISO-stimulated Ca_V_1.2 superclustering response persists in cultured myocytes despite an overall reduction in Ca_V_1.2 channel cluster size and expression compared to uncultured cells (compare Fig. 5 *A*–*C* to Fig. 3 *A*–*C*). Whole-cell patch clamp recordings of RFP transduced control cells further confirmed that *β*-adrenergic regulation of *I*_Ca_ was present in these cells with ISO eliciting a 1.73 ± 0.21-fold increase in peak current density (Fig. 5 *D* and *E*), and a significant leftward-shift and steeper slope in the voltage dependence of conductance (Fig. 5*F* and *SI Appendix,* Table S2) as expected. Overexpression of 14-3-3ε produced an increase in basal Ca_V_1.2 channel cluster size but no increase in the mean gray value suggesting channel expression was not altered by 14-3-3ε overexpression compared to RFP-transduced controls (Fig. 5 *A*–*C*). Supporting that idea, basal whole-cell current density was unaltered by 14-3-3ε overexpression (Fig. 5 *D* and *E*) and cell-attached patch recordings revealed no significant differences in basal Ca_V_1.2 single-channel activity compared to RFP transduced controls (*SI Appendix,* Fig. S9). Similarly, difopein-mediated 14-3-3 inhibition had no significant impact on basal Ca_V_1.2 channel clustering, expression, whole-cell Ca^2+^ currents, or single-channel activity (Fig. 5 *A*–*F* and *SI Appendix,* Fig. S9). However, 14-3-3 manipulation significantly impacted *β*-adrenergic regulation of Ca_V_1.2 channels, with 14-3-3ε overexpression augmenting and difopein inhibition attenuating both ISO-stimulated superclustering and *I*_Ca_ enhancement (Fig. 5 *A*–*E*). These more prolonged effects of 14-3-3 inhibition mirror the acute effects observed with BV02 (Fig. 4). Unfortunately, we were unable to maintain stable cell-attached seals for the duration needed to record both control and ISO-stimulated single-channel activity in cultured cardiomyocytes. However, based on our observations of altered superclustering and *I*_Ca_, we anticipate that 14-3-3’s effects on *β*-adrenergic regulation of Ca_V_1.2 channels should also be detectable at the single-channel level. Interestingly, difopein-transduced myocytes continued to display an ISO-stimulated leftward-shift with a similar V_1/2_ despite the loss of peak current enhancement and channel superclustering (*SI Appendix,* Table S2). These data reveal that 14-3-3 is a critical player in *β*-adrenergic regulation of Ca_V_1.2 recycling, superclustering, and the associated *I*_Ca_ enhancement in cardiomyocytes.

### 14-3-3 Overexpression or Prolonged Inhibition Alters Ca^2+^ Handling and EC-Coupling.

Since Ca^2+^-influx through Ca_V_1.2 channels is the trigger for CICR from RyR2 during EC-coupling, we hypothesized that these effects of 14-3-3 on *β*-adrenergic regulation of Ca_V_1.2 would also impact the heart’s ability to tune EC-coupling. Thus, to investigate the contribution of 14-3-3 to cardiac Ca^2+^ handling and EC-coupling we paced Rhod2-AM loaded cardiomyocytes from each of the RFP, 14-3-3ε, and difopein transduced cultured cell cohorts at a frequency of 1 Hz and performed line-scan confocal imaging across the length of individual cells to record Ca^2+^ transients before and after ISO. Consistent with our SMLM imaging and electrophysiological data, control (no-ISO) Ca^2+^ transient amplitudes remained unchanged whether cells were transduced with 14-3-3ε, difopein, or RFP (Fig. 5 *G*–*I*). In contrast, control transient decay kinetics were significantly slower in 14-3-3ε overexpressing cells compared to RFP or difopein transduced myocytes (Fig. 5 *J* and *K*), suggesting 14-3-3ε decreases the efficiency of Ca^2+^ extrusion mechanisms. However, during *β*-adrenergic stimulation, 14-3-3ε overexpressing cells exhibited a more pronounced acceleration of their transient decay kinetics compared to RFP or difopein transduced myocytes. 14-3-3 inhibition with difopein diminished the positive inotropic response to ISO (measured by Ca^2+^ transient amplitude), while 14-3-3ε overexpression enhanced it. These findings suggest that 14-3-3 impacts Ca^2+^ handling and regulation of cardiac EC-coupling during *β*-adrenergic stimulation.

## Discussion

We demonstrate a regulatory mechanism for Ca_V_1.2 through 14-3-3 interactions, supported by multiple lines of evidence: 1) Ca_V_1.2 and 14-3-3 form complexes in tsA-201 cells; 2) Airyscan imaging and PLA show increased 14-3-3/Ca_V_1.2 association in cardiomyocyte t-tubules following *β*-adrenergic stimulation; 3) superresolution imaging reveals 14-3-3 regulates Ca_V_1.2 nanoscale distribution during *β*-adrenergic signaling; 4) 14-3-3 facilitates Ca_V_1.2 trafficking in tsA-201 cells; 5) 14-3-3 modulates *β*-adrenergic regulation of Ca_V_1.2 recycling, superclustering, and *I*_Ca_ enhancement in cardiomyocytes; and 6) 14-3-3 affects the efficiency of Ca^2+^ extrusion mechanisms and influences the inotropic response during *β*-adrenergic regulation of cardiac EC-coupling.

14-3-3 is known to interact with many ion channels and has recently been appreciated as an important regulator of many EC-coupling proteins. In the heart, 14-3-3 regulates Na_V_1.5 (20, 21), K^+^ channels such as hERG (18), TASK-1 (19) and K_ATP_ (15), and Ca^2+^ handling proteins such as SERCA through phospholamban (23), PMCA (24, 25), and NCX (26). In the brain and in heterologous expression systems, 14-3-3 also regulates voltage-gated N-type Ca^2+^ channels (Ca_V_2.2), a structurally similar calcium channel involved in neurotransmitter release (27, 28). Despite these discoveries, there has been no direct investigation of Ca_V_1.2 regulation by 14-3-3. A study pursuing novel approaches to inhibit calcium channels found that artificially membrane-bound 14-3-3ε was able to inhibit Ca_V_1.2, presumably by binding to the channel and pulling a cytosolic portion of the channel into an unfavorable position (29). Although this study showed an interaction between the modified 14-3-3ε and Ca_V_1.2, the endogenous effects of 14-3-3 on Ca_V_1.2 were not examined. Our study provides evidence for complex formation between 14-3-3 and Ca_V_1.2, both in heterologous systems and cardiac myocytes.

Our results support a role for 14-3-3 in regulation of Ca_V_1.2 channel expression at the plasma membrane. In tsA-201 cells, 14-3-3ε overexpression promoted enhanced expression of Ca_V_1.2 at the plasma membrane while competitive inhibition of 14-3-3 had the opposite effect, resulting in a near total inability of the channels to get to the membrane. This dependence of Ca_V_1.2 trafficking on 14-3-3 in tsA-201 cells is supported by both imaging and electrophysiology experiments in which *I*_Ca_ and single-channel activity were extremely low in cells transfected with difopein compared to controls transfected with or without inactive difopein. In that series of experiments, it was difficult to determine whether a patched cell displayed no current because some element of the channel complex had not transfected adequately or because of an effect of difopein; thus, a criterion for inclusion in the analysis was that the cell must have some measurable Ca^2+^ current. This level of caution likely means we are underestimating the effect of 14-3-3 inhibition. Our findings are consistent with the report that 14-3-3τ promotes plasma membrane localization of voltage-gated N-type Ca_V_2.2 Ca^2+^ channels in tsA-201 cells (28). Although tsA-201 cells endogenously express all transcripts of 14-3-3 isoforms (57), our results indicate that endogenous or even overexpressed 14-3-3 is not sufficient to drive membrane expression of Ca_V_1.2 channels in the absence of auxiliary subunits, unlike for Ca_V_2.2. Rather it seems that 14-3-3 acts in concert with the auxiliary subunits to promote membrane expression of Ca_V_1.2 α_1C_. It is generally accepted that Ca_V_β subunits are required for trafficking of α_1C_ however a recent study reported that cardiac α_1C_ can traffic to the sarcolemma of cardiomyocytes even when they have a mutation that abrogates their binding to Ca_V_β subunits (58). While interpretation of those results is complicated by the fact that the mutant channel trafficking was studied in cells that also expressed WT channels that could bind endogenously expressed Ca_V_β subunits, it remains an untested possibility that endogenous 14-3-3 could facilitate forward trafficking of those mutant channels in cardiomyocytes.

One of the original hypotheses as to how Ca_V_β could facilitate Ca_V_1.2 trafficking was that its binding to the AID region of the I-II loop of the channel masked a unidentified ER-retention motif (59). That motif was never identified and the hypothesis was largely rejected (60, 61) with subsequent work revealing that the proximal C-terminus of Ca_V_1.2, far from the AID, is critical for membrane targeting of α_1C_ (62). CD4-fusion constructs of various segments of the Ca_V_1.2 α_1C_ revealed two putative ER-retention motifs in the proximal C-terminal of Ca_V_1.2, and one in a similar region of Ca_V_2.2 (61). A similar approach using Myc-CD8α fused to segments of the C-terminal of rat Ca_V_2.2 confirmed that amino acids 1706-1940 on the proximal C-terminal contains an ER-retention signal that can be masked by 14-3-3 binding to allow surface expression of the channel (28). A second phospho-regulated 14-3-3 binding site on the distal Ca_V_2.2 C-terminal (S2126) was found to affect the inactivation properties of the channel, although they did not observe changes to Ca_V_1.2 inactivation in the same conditions (27, 28). In line with these results, we did not observe any effects of 14-3-3 on Ca_V_1.2 inactivation properties, however mapping of the possible 14-3-3 binding sites predicted five phosphorylation-dependent 14-3-3 binding sites on the C-terminal tail of Ca_V_1.2, in addition to the C-terminal site. Of these five, two are in similar positions to the sites found on Ca_V_2.2, one located proximally (S1700), and another located distally (S1928). Interestingly, mutant animals with phosphorylation-preventing alanine substitutions of S1700 and T1704 on the C-tail of Ca_V_1.2 display significantly suppressed sarcolemmal expression of the channel and a correspondingly reduced basal *I*_Ca_ but intact responsivity to *β*-AR stimulation (63). A similar mutant mouse generated independently displayed a comparably reduced basal *I*_Ca_ but was said to have similar expression levels of the channel; however, a definitive biotinylation-assay was not performed to quantify the membrane population (64). It is possible that 14-3-3 binding to phosphorylated S1700 or perhaps T1704 masks an ER-retention signal located in that proximal segment of the C-terminal tail, facilitating surface expression of the channels. In contrast, phosphorylation of the S1928 site has been associated with PKA-dependent acute increases in Ca_V_1.2 activity in vascular smooth muscle (65) and neurons (66). Although this site is a PKA target in cardiac Ca_V_1.2 as well, there has been no such definitive regulatory role for S1928 phosphorylation in the heart (67). Binding of 14-3-3 to both sites simultaneously may allow phosphorylation-dependent trafficking of Ca_V_1.2 based on the channel’s proximity to *β*-adrenergic signaling complexes. AF3 modeling predicted a potential structure of Ca_V_1.2 with 14-3-3 bound concurrently at S1700 and S1928. However, AF3 also predicts 14-3-3ε can bind to at least two other phosphorylated components of the Ca_V_1.2 channel complex, namely Ca_V_β and the RGK-protein Rad. These models illustrate that there are multiple possible sites of interaction between 14-3-3ε and Ca_V_1.2 channel complex. A future interesting question to pursue on this point will be to determine whether 14-3-3ε binding to Ca_V_β and/or to Rad obstructs or competitively inhibits the Ca_V_β–Rad interaction. It took over 40 y to definitively identify the critical PKA-phospho-site on the Ca_V_1.2 complex, one hopes it will not take so long to determine the critical Ca_V_1.2–14-3-3 interaction site; this should be an important avenue for future research.

Our findings imply that the surface expression of Ca_V_1.2 is promoted by their phosphorylation-dependent association with 14-3-3. Phosphorylation-dependent anterograde trafficking of ion channels has been proposed for several K^+^ channels (15, 19, 68), Na^+^ channels (69), and Ca_V_1.2 Ca^2+^ channels (63, 70). In the case of TASK potassium channels the underlying mechanism has been well studied and involves phosphorylation and 14-3-3-binding acting as a “release” switch that prevents an otherwise constitutive binding of the channels to coat protein (COP) complex I (COPI) (19, 71). COPI mediates retrograde transport of proteins from endosomes back to the Golgi and from the Golgi back to the ER but phosphorylation of TASK-3 and binding of 14-3-3 has been proposed to release the channels from COPI to allow their anterograde transport and membrane expression. Cardiac ATP-sensitive potassium channels have been found to display a similar increase in sarcolemmal expression in response to their phosphorylation downstream of *β*-AR stimulation (15). Furthermore, previous work from our group revealed that endosomal reservoirs of Ca_V_1.2 channels are recycled back to the membrane in a phosphorylation-dependent manner upon *β*-AR stimulation (45–48). These prior results from our group and others, paired with the findings in the present study, and our AF3-predicted model of association between Ca_V_1.2 and 14-3-3ε invite the speculative conclusion that a similar mechanism could be in place for these voltage-gated Ca^2+^ channels.

In cardiomyocytes inhibition of 14-3-3 with either difopein or BV02 did not overtly alter basal *I*_Ca_ or channel expression in-so-far as we can tell from imaging experiments. Since both difopein and BV02 are competitive inhibitors of 14-3-3 it may be that we are not achieving high enough concentrations of these agents to have the desired dominant negative effect. It may also be a case of bad timing in that channels may require 14-3-3 to bind to them during their transit through the biosynthetic delivery pathway, thus the effects may be easier to observe on the blank slate of a tsA-201 cell where we can manipulate 14-3-3 levels/binding while the channels are all actively being translated. Thus, effects of 14-3-3 on basal expression may be more difficult to ascertain in ventricular myocytes. However, it is clear that 14-3-3 plays a role in modulating the response to *β*-AR stimulation with 14-3-3 inhibition abrogating the ISO-induced superclustering of Ca_V_1.2 channels and significantly blunting the *I*_Ca_ augmentation. Interestingly, although the increase in *I*_Ca_ was abolished with 14-3-3 inhibition, we still observed a significant left-shift in voltage dependence of activation. This suggests that although both effects are PKA dependent, they are occurring through separate mechanisms, only one of which is regulated by 14-3-3.

The effect of 14-3-3 on ISO-stimulated Ca_V_1.2 channel superclustering has implications for cooperative gating. This gating modality has been proposed in multiple ion channels where physical interactions between adjacent channels allow allosteric communication such that the opening of one channel in a cluster can increase the probability of gating of the adherent channels (38). Ca_V_1.2 channels cooperatively gate to enhance Ca^2+^ influx (37, 39), promoted by Ca_V_1.2 supercluster formation during *β*-AR activation (45, 46). This process is calmodulin-dependent, but since 14-3-3 can link Na_V_1.5 channels, we hypothesized 14-3-3 might facilitate Ca_V_1.2 cooperative gating by linking channels into larger oligomers, a function observed in plants and mammals (21, 72). While oligomerization was not directly tested, multi-Ca_V_1.2 channel openings increased in 14-3-3ε overexpressing tsA-201 cells, with cooperativity measures showing positive 14-3-3 effects and negative difopein effects. These suggestive findings need further study, as our data do not definitively link 14-3-3 to Ca_V_1.2 oligomerization beyond the observation that it regulates cluster size and channel expression on the plasma membrane. In cardiomyocytes, 14-3-3’s effect on Ca_V_1.2 cooperativity was not apparent, although its role in nucleating Ca_V_1.2 superclustering during *β*-AR signaling could mean enhanced channel cooperativity would become evident following ISO stimulation.

Our findings also demonstrated that 14-3-3 manipulation significantly influenced *β*-AR-stimulation of EC-coupling. Specifically, difopein-mediated inhibition attenuated ISO-stimulated enhancement of Ca^2+^ transient amplitudes, while 14-3-3ε overexpression potentiated them. These results parallel our electrophysiology data, where both difopein-transduced myocytes and acute 14-3-3 inhibition with BV02 significantly reduced ISO’s *I*_Ca_-enhancing effects, with 14-3-3ε overexpression producing the opposite effect. This relationship can be explained by the mechanistic link between Ca_V_1.2 channel-mediated Ca^2+^ influx and the subsequent graded release of Ca^2+^ from the SR, which ultimately determines both Ca^2+^ transient amplitudes and contractile force. Particularly intriguing were the effects of 14-3-3 manipulation on transient decay kinetics. 14-3-3ε overexpression produced two seemingly contradictory effects: a slowing of decay kinetics under basal conditions but an enhancement of ISO-stimulated decay acceleration. This apparent paradox suggests that 14-3-3ε exerts both phosphorylation-independent and phosphorylation-dependent effects on Ca^2+^ extruding proteins. The basal slowing of Ca^2+^ decay rates can be attributed to 14-3-3ε’s documented interactions with NCX2 and PMCA4, where it inhibits their Ca^2+^ extrusion capabilities independent of phosphorylation status (25, 26). In contrast, during *β*-adrenergic stimulation, 14-3-3’s binding to phosphorylated phospholamban prevents its dephosphorylation, thereby maintaining SERCA in an activated state (23). This dual mechanism explains the enhanced ISO-stimulated acceleration of transient decay rates in 14-3-3ε overexpressing cells. Although we did not measure SR Ca^2+^ load, the sustained SERCA activation likely increases SR Ca^2+^ content, potentially contributing to the amplified Ca^2+^ transients observed in these cells. Collectively, these findings reveal 14-3-3 proteins as sophisticated regulators of cardiac Ca^2+^ handling, capable of modulating multiple aspects of EC-coupling through both phosphorylation-dependent and -independent mechanisms.

Our findings establish a paradigm wherein 14-3-3ε emerges as a critical modulatory protein of Ca_V_1.2 channels, orchestrating channel trafficking, clustering, and *β*-AR signal responsiveness through phosphorylation-dependent interactions. The data support a model where 14-3-3 serves as a key nucleation factor facilitating Ca_V_1.2 recycling into the sarcolemma during *β*-AR stimulation (*SI Appendix,* Fig. S10). These findings not only expand the growing network of EC-coupling proteins under 14-3-3 regulation but also provide crucial mechanistic insights into the dynamic control of cardiac calcium channels. Understanding these regulatory pathways may reveal therapeutic strategies for modulating cardiac contractility in both physiological and pathological states.

## Materials and Methods

All animal handling and procedures adhered to the NIH Guide for the Care and Use of Laboratory Animals (UC Davis) and were approved by the local Institutional Animal Care and Use Committee. tsA-201 cells were transiently transfected with combinations of Ca_V_1.2 α_1C_, Ca_V_β_2b_, Ca_V_α_2_δ, 14-3-3 isoforms, difopein, or inactivated difopein and used for microscopy and patch clamp electrophysiology experiments. Ventricular myocytes were isolated from 3 to 6-mo-old C57BL/6J mouse hearts via retrograde Langendorff perfusion as previously described (45, 46) and used in microscopy and patch clamp electrophysiology experiments. *N* represents the number of animals and *n* represents the number of cells. Data are reported as mean ± SEM. Statistics were performed using Prism (GraphPad Software Inc.), and all datasets were tested for normality. Unpaired Student’s *t* tests or Mann–Whitney tests were used to compare datasets with two groups, one-way ANOVAs or Kruskal–Wallis (nonparametric) tests with post hoc testing were used to compare datasets with more than two groups, or two-way ANOVAs when there were two independent variables. *P* < 0.05 was considered statistically significant. Detailed methods can be found in *SI Appendix*.

## Supplementary Material

Appendix 01 (PDF)

Dataset S01 (XLSX)

## Data Availability

All study data are included in the article and/or supporting information.
